# Unwise Laws

**Published:** 1887-09

**Authors:** 


UNWISE LAWS: a Consideration of the Operations of a Protective Tariff, upon Industry, Commerce and Society. By Lewis H. Blair, of Richmond, Va. Published by G. P. Putnam's Sons. The Knickerbocker Press. London and New York, 1886. Cloth, 177 pages.

This kind of literature is a little out of the line of medical journalism; but when we consider that many physicians are either pecuniarily interested in the commerce of the country, or are politicians enough to take some interest in the tariff, we are justified in mentioning this work here: especially as it is written by a man who is, himself, a merchant, and who discusses the subject in a common sense way,—not from the standpoint of any particular industry to be affected one way or the other, by tariff laws, but on general principles. The author says, the book "is intended for plain sensible people, who have no time or taste for elaborate disquisitions on the tariff, but who nevertheless, would be glad to know something about the subject;" and, while his effort, he says, "may not be equal to an incandescent lamp, it is yet clear and strong enough, he hopes, to penetrate and scatter the mists that have been purposely thrown, by designing men, around the subject of protection, and to lead his readers to his own conclusions, that all taxes should be exclusively for benefit of government and none for benefit of industries, whether infant or ancient."

From the authors standpoint, there is evidently, "something rotten in Denmark."

